# Captivity Reduces Diversity and Shifts Composition of the Great Bustard (*Otis tarda dybowskii*) Microbiome

**DOI:** 10.1002/ece3.70836

**Published:** 2025-01-10

**Authors:** Zhiyuan Lu, Zhucheng Wang, Hexue Jia, Derong Meng, Dayong Wu

**Affiliations:** ^1^ College of Life Sciences Cangzhou Normal University Cangzhou China; ^2^ Collaborative Innovation Center for Wetland Conservation and Green Development of Hebei Province Hengshui University Hengshui China; ^3^ Center for Wetland Conservation and Research Hengshui University Hengshui China; ^4^ Hebei Key Laboratory of Wetland Ecology and Conservation Hengshui China

**Keywords:** conservation, great bustard, gut microbiota, high‐throughput sequencing

## Abstract

Captivity offers protection for endangered species, but for bustards, captive individuals face a higher risk of disease and exhibit lower reintroduction success rates. Changes in the diversity of host bacterial and fungal microbiota may be a significant factor influencing reintroduction success. The great bustard ( *Otis tarda* ) is a globally recognized endangered bird species. Previous research on the gut microbiota of the great bustard has been limited, hindering effective conservation efforts. Therefore, this study utilized high‐throughput sequencing of the 16S rRNA and Internal Transcribed Spacer (ITS) genes to compare the gut bacterial and fungal microbiota of great bustards in different environments. The results revealed a significant decline in alpha diversity and notable changes in microbial community structure in captive environments. Changes in diet and habitat are likely major factors contributing to these shifts. Consequently, managing rescued wild animals by increasing dietary diversity and exposure to natural environmental reservoirs may enhance the success rate of reintroduction efforts.

## Graphical abstract

This study investigates the impact of captive environments on the gut microbiota of endangered great bustards ( *Otis tarda* ), crucial for successful reintroduction efforts. Through high‐throughput sequencing, it identifies significant declines in alpha diversity and shifts in microbial community structure in captive environments, attributed to changes in diet and habitat. The findings underscore the importance of managing rescued wild animals by enhancing dietary diversity and exposure to natural environments to bolster reintroduction success.

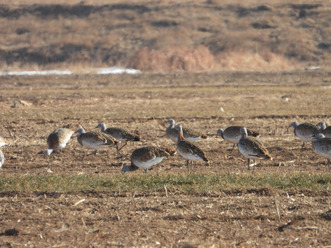

## 1 Introduction

Throughout the extensive history of agricultural development, numerous wild bird species have adapted to or even become reliant on farmland habitats. However, the rapid expansion and intensification of modern agricultural practices are now significant drivers of biodiversity loss (Kehoe et al. 2017; Li et al. 2020). Among the many species under threat, the great bustard ( *Otis tarda* ) stands out as a farmland‐dependent bird that has attracted substantial concern regarding its conservation status (Liu et al. 2022; Lu et al. 2021). The great bustard is one of the heaviest flying birds in existence and functions as an indicator species for grassland ecosystems. It is categorized as Endangered on the International Union for Conservation of Nature (IUCN) Red List and is designated as a Class I nationally protected species in China (Dunning Jr 2007; Li, Liu, and Tian 2017; Santana et al. 2014). The species faces severe challenges due to habitat destruction, climate change, and illegal hunting, which have all contributed to the decline of its natural populations (Alonso and Palacín 2022; Collar et al. 2017). The great bustard comprises two subspecies: the nominal subspecies ( *O. tarda tarda* ) and the Asian subspecies ( *O. tarda dybowskii* ). The Asian subspecies is primarily distributed in East Asia, including Russia, Mongolia, and China (Kessler et al. 2013). This subspecies represents less than 10% of the global population and is at a high risk of extinction (Liu et al. 2017).

To address this challenge, captive breeding, and reintroduction programs have become crucial strategies for the conservation of endangered bird species (Badia‐Boher et al. 2022; Dolman et al. 2021; Tripovich et al. 2021). However, these programs have encountered significant setbacks in the conservation of bustards. The outcomes of a 10‐year reintroduction trial of great bustards in the UK were highly discouraging, with only 11.3% of artificially hatched chicks surviving beyond 1 year after release (Ashbrook et al. 2016). From 1988 to 2008, researchers investigated 198 Kori bustards housed in 36 U.S. zoos and found that captive Kori bustards had a 93% probability of developing disease, with trauma, infections, and parasites identified as common contributing factors (Hanselmann et al. 2013). There are significant differences between captive and natural environments, which can have profound effects on the physiology and behavior of animals (Bussolini et al. 2023; Checon et al. 2020). These differences are especially impactful on the gut microbiome, a complex biological system (Alberdi, Martin Bideguren, and Aizpurua 2021; Oliveira et al. 2020; Xie et al. 2016). In captive environments, the gut microbial communities of birds may be influenced by various factors such as dietary changes, restricted physical space, reduced social interactions, and increased environmental stress (Dallas and Warne 2023). These changes can lead to alterations in the gut microbiota, which in turn can affect the health of the birds (Naz et al. 2020; Song et al. 2022). For instance, the high stress and limited space in captive environments may cause an overgrowth of certain pathogens, triggering inflammatory responses (Craft et al. 2022; Madden et al. 2022). Furthermore, the non‐natural environment can disrupt the balance of gut microbiota, impacting the birds' digestive capabilities and immune functions (Bodawatta et al. 2021a; Van Veelen et al. 2020).

Bacterial and fungal microbiomes are integral components of the avian digestive system, engaging in various biological processes such as food digestion, nutrient absorption, immune system regulation, and pathogen resistance (Aruwa et al. 2021; Bodawatta et al. 2022; Elliott et al. 2019; Grond et al. 2018). Beneficial bacterial communities, in particular, aid the host in efficiently breaking down complex carbohydrates in food and synthesizing short‐chain fatty acids (Biddle et al. 2013; Kaoutari et al. 2013). Furthermore, they interact with the host's immune system to help prevent pathogen invasion and colonization (Luo et al. 2020; Xing et al. 2019). Fungal microbiomes also play a crucial role in maintaining microbial balance and promoting host health (Elliott et al. 2019; Roto, Rubinelli, and Ricke 2015). Research indicates that fungal microorganisms can regulate host fat metabolism, facilitate the degradation of cellulose and other carbohydrates, initiate immune pathways, and modulate inflammatory responses (Heisel et al. 2017; Yang et al. 2018; Yeung et al. 2020). Additionally, certain fungal metabolites have antiparasitic properties, protecting the host from intestinal infections (Lozano et al. 2024).

The gut microbiota of wild birds have been extensively characterized through the advanced application of high‐throughput sequencing technologies (Bodawatta et al. 2021b; Hird et al. 2015; Laviad‐Shitrit et al. 2019). In the conservation of endangered birds, captivity, as a key conservation strategy, often alters the host's gut microbiota, which may subsequently reduce the success of reintroduction efforts (Trevelline et al. 2019; West et al. 2019). In the endangered western capercaillie ( *Tetrao urogallus* ), captive individuals show an increased richness of bacterial taxa associated with intestinal dysfunction, which has been proposed as a contributing factor to unsuccessful reintroductions (Wienemann et al. 2011). Integrating wildlife conservation efforts with microbiome research is becoming increasingly important (Dallas and Warne 2023). The great bustard is an endangered bird species of global concern, and our understanding of its gut microbiota is extremely limited. The limited studies available have primarily focused on describing the gut bacterial microbiota in wild populations, while information on gut fungal communities remains scarce (Liu et al. 2020; Lu et al. 2022). Furthermore, there are no published reports on the gut microbiota of captive great bustards. Therefore, this study utilizes high‐throughput sequencing of the 16S rRNA gene and the Internal Transcribed Spacer (ITS) gene to compare the differences in gut bacterial and fungal microbiomes of great bustards across different environments. This approach aims to elucidate the impact of captive environments on the gut microbiota of the great bustard, thereby providing a foundation for the development of more effective conservation and management strategies.

## 2 Materials and Methods

### 2.1 Sample Collection

The Cangzhou region in China is a crucial wintering and stopover site for approximately 300 individuals of the Asian great bustard annually (Lu et al. 2024; Mi et al. 2017). The Cangzhou Wildlife Rescue Center is dedicated to the rescue and rehabilitation of local wildlife, with a particular focus on the great bustard. In January 2020, 13 fresh fecal samples (W1–W13) were collected from a stable wintering habitat of wild great bustards. In December 2019, four fecal samples (C1–C4) were collected from captive great bustards at the Cangzhou Wildlife Rescue Center. Four captive great bustards, all rescued from the wild, including two females and two sub‐adults of unknown sex, were housed together in an outdoor rehabilitation enclosure due to their inability to survive in the wild. They were fed a daily diet consisting of market‐purchased soybeans and peanuts.

**FIGURE 1:**
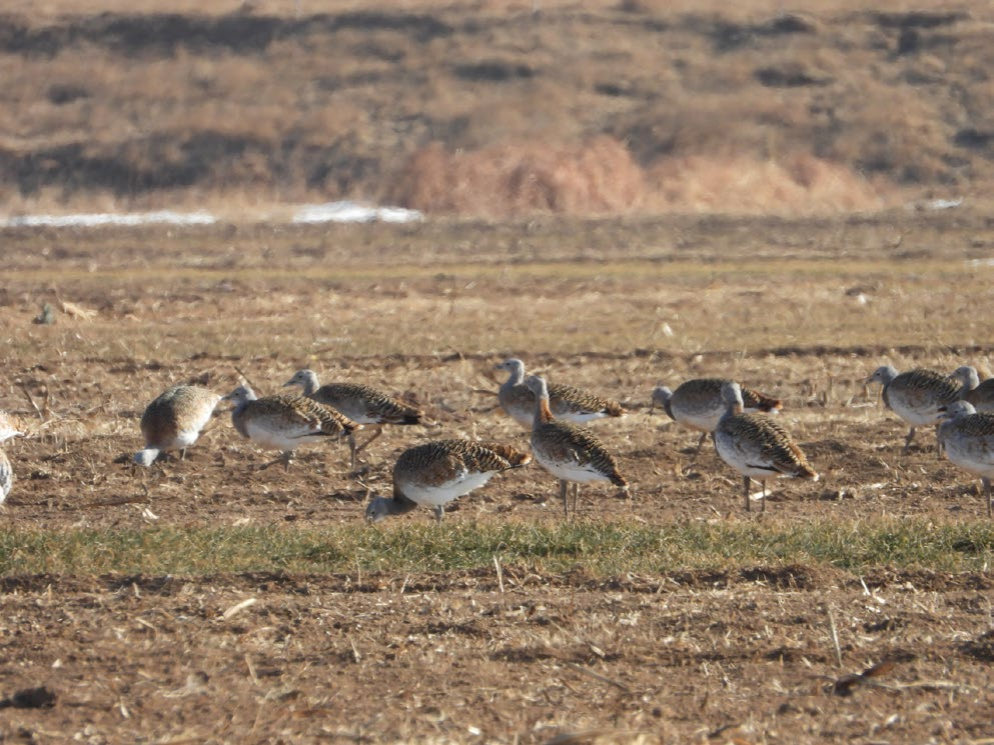
The great bustards foraging in the wild.

The overwintering populations of wild great bustards typically move between multiple stable habitats, with recorded group sizes ranging from 13 to 64 individuals (Figure 1). Using high‐power monocular telescopes, we tracked the wild great bustard populations, selecting groups with more than 20 individuals and ensuring no other species were mixed. After the group finished foraging and flew away, we used disposable sterile tweezers to collect fresh fecal samples that appeared moist from the ground. To avoid sampling the same individual multiple times, we maintained a sampling distance of at least 5 m. The non‐environmental contact portion of the feces was extracted and placed in sterile centrifuge tubes, then transported back to the laboratory in a portable car refrigerator. In the rehabilitation shed of the Cangzhou Wildlife Rescue Center, the defecation behavior of captive great bustards was observed via video surveillance to collect fecal samples from different individuals. All fecal samples were stored in an ultra‐low temperature freezer at −80°C.

### 2.2 Sample DNA Extraction, Amplification, and Sequencing

Total DNA was extracted from the samples using the E.Z.N.A. Soil DNA Kit (Omega Bio‐Tek, USA) following the manufacturer's instructions. The concentration and purity of the DNA were measured with a NanoDrop 2000 spectrophotometer (Thermo Fisher Scientific, USA), and the quality of the DNA extraction was further verified by 1% agarose gel electrophoresis. The hypervariable V3‐V4 region of the bacterial 16S rRNA gene was amplified using primers 338 F (5′‐ACTCCTACGGGAGGCAGCAG‐3′) and 806 R (5′‐GGACTACHVGGGTWTCTAAT‐3′) (Mori et al. 2014). For fungal ITS gene amplification, primers ITS 1F (5′‐CTTGGTCATTTAGAGGAAGTAA‐3′) and ITS 2R (5′‐GCTGCGTTCTTCATCGATGC‐3′) were used to target the ITS1 region (Adams et al. 2013). The PCR reaction mixture consisted of 4 μL of 5× FastPfu buffer, 2 μL of 2.5 mM dNTPs, 0.8 μL of forward primer (5 μM), 0.8 μL of reverse primer (5 μM), 0.4 μL of FastPfu polymerase, 10 μL of DNA template (1 ng/μL), and ddH2O added to a final volume of 20 μL. The PCR conditions were as follows: initial denaturation at 95°C for 3 min, followed by 27 cycles of 95°C for 30 s, 55°C for 30 s, and 72°C for 45 s, with a final extension at 72°C for 10 min. The PCR products from three replicates were combined in equimolar concentrations and analyzed using 2% agarose gel electrophoresis. The products were purified with the AxyPrep DNA Gel Extraction Kit (Axygen Biosciences, USA) and quantified using a QuantiFluor‐ST fluorometer (Promega, USA). Finally, a paired‐end (PE) 2 × 300 library was constructed and sequenced on the MiSeq PE300 platform (Illumina, USA).

### 2.3 Data Processing and Analysis

The raw sequences were processed following the standard procedures of QIIME 2 (Bolyen et al. 2019). After demultiplexing the raw data by sample, quality control was performed using Fastp v0.20.0 (Chen et al. 2018), and sequences were merged with FLASH v1.2.7 (Magoc and Salzberg 2011). The data were then processed with DADA2 to obtain amplicon sequence variants (ASVs) (Callahan et al. 2016). For bacterial ASVs classification and annotation, a naive Bayes classifier was used with a 70% confidence threshold against the Silva 16S rRNA database (SSU138) (Quast et al. 2012), excluding chloroplast and mitochondrial ASVs. Fungal ASVs were classifiedand annotated using the UNITE database (Abarenkov et al. 2010).

Alpha diversity indices based on ASVs, including community richness (Sobs), evenness (Shannoneven), diversity (Shannon), and coverage (Coverage), were calculated using Mothur v1.30.2. Differences in alpha diversity indices between groups were analyzed using the Mann–Whitney *U* test in R package stats. The *p*‐values were further adjusted for multiple comparisons using the False Discovery Rate (FDR) method. Rarefaction curves for the Sobs index of each sample group were generated using the Mothur‐1.30.2 software to assess the adequacy of sequencing data. Venn diagrams were utilized by the R package stats to identify shared and unique ASVs among groups. To visualize community composition at different taxonomic levels, bar charts, and heatmaps were created by the R package pheatmap. The abund_jaccard‐distance based principal coordinate analysis (PCoA) was performed using r package vegan to evaluate the variability of gut microbiota between different sample groups. The statistical significance of this variability was assessed using Permutational Multivariate Analysis of Variance (PERMANOVA). Linear Discriminant Analysis (LDA) Effect Size (LEfSe) on the Galaxy platform was used to identify microbial taxa with statistically significant differences in abundance between groups (Segata et al. 2011). Functional predictions and abundance information for ASVs in each sample were inferred using PICRUSt2 based on the KEGG database. Differences in the functional abundance of samples between groups were tested using the Mann–Whitney *U* test in R package stats. The *p*‐values were further adjusted for multiple comparisons using the False Discovery Rate (FDR) method.

## 3 Results

### 3.1 Sequencing Analysis

DNA was extracted from the samples, followed by the amplification and sequencing of the 16S rRNA and ITS genes. After assembling and quality controlling the raw sequences, a total of 849,873 filtered 16S rRNA gene sequences were obtained, with individual sequence counts ranging from 40,785 to 62,311 (Table S1). For the ITS gene, a total of 1,151,766 filtered sequences were obtained, with counts ranging from 57,305 to 81,260 (Table S2). These optimized sequences underwent denoising and were subjected to secondary sampling. To avoid statistical discrepancies caused by varying sequencing depths, random resampling was performed using the minimum sample sequence count. The minimum number of sequences for the bacterial samples is 20,009, while for the fungal samples, it is 50,646. After annotation, 1308 ASVs were identified from the 16S rRNA gene sequences, distributed across 13 phyla, 20 classes, 60 orders, 93 families, and 202 genera. Similarly, 1075 ASVs were identified from the ITS gene sequences, distributed across 7 phyla, 24 classes, 65 orders, 149 families, and 274 genera. The rarefaction curves indicated that the sequencing depth was sufficient to capture the majority of microbial diversity present in all samples (Figure S1).

### 3.2 Microbial Composition and Relative Abundance

**FIGURE 2:**
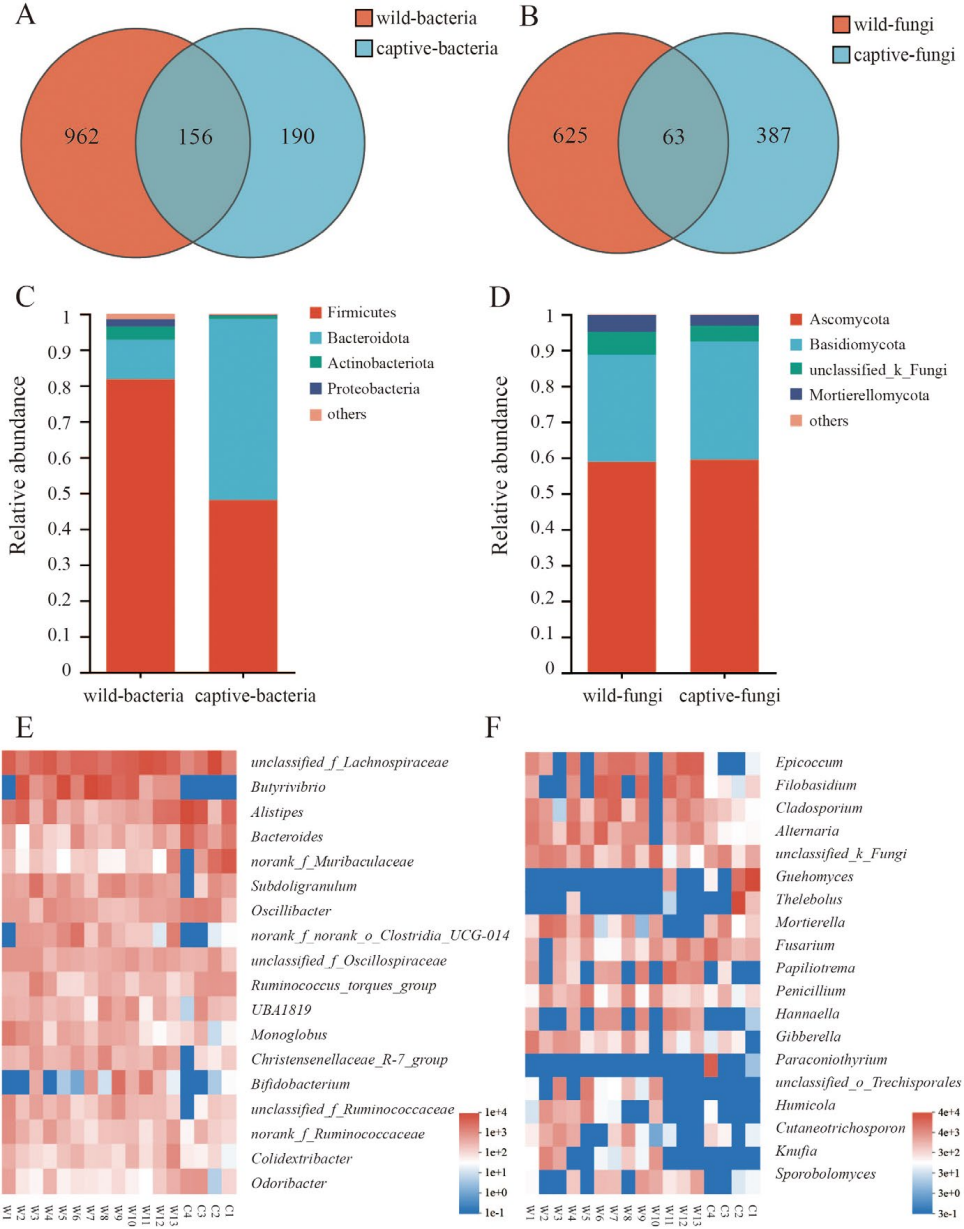
(A) The Venn diagram of bacterial ASVs between the two sample groups. (B) The Venn diagram of fungal ASVs between the two sample groups. (C) The relative abundance of bacterial microbiota at the phylum level. (D) The relative abundance of fungal microbiota at the phylum level. (E) The heatmap displays the bacterial microbiota structure of core genera across all samples. (F) The heatmap displays the fungal microbiota structure of core genera across all samples.

Core microbial composition (mean relative abundance > 1%) was determined by analyzing the gut microbiota of overwintering great bustards. In the field sample set, the dominant bacterial taxa were Firmicutes (81.84% ± 7.79%), Bacteroidota (10.97% ± 7.84%), Actinobacteriota (3.71% ± 4.06%), and Proteobacteria (2.03% ± 3.38%), collectively accounting for 98.55% ± 1.45% of the total microbial abundance across all samples (Figure 2C). In the caged sample group, the dominant bacterial taxa were Firmicutes (48.19% ± 21.77%), Bacteroidota (50.34% ± 22.9%), and Actinobacteriota (1.02% ± 1.06%), together representing 99.55% ± 0.19% of the total microbial abundance (Figure 2C). Additionally, 18 core bacterial genera were identified across all samples, contributing to 79.56% ± 7% of the total microbial composition (Figure 2E).

At the fungal phylum level, the dominant taxa in the field sample set were Ascomycota (58.89% ± 14.05%), Basidiomycota (29.92% ± 18.16%), unclassified Fungi (6.26% ± 6.73%), and Mortierellomycota (4.81% ± 6.9%), which together accounted for 99.88% ± 0.32% of the total fungal abundance (Figure 2D). In the caged sample group, the dominant fungal taxa were Ascomycota (59.49% ± 27.8%), Basidiomycota (33% ± 28.78%), unclassified Fungi (4.34% ± 3.78%), and Mortierellomycota (3.08% ± 4.35%), representing 99.91% ± 0.13% of the total fungal abundance (Figure 2D). Additionally, 19 core fungal genera were identified across all samples, comprising 73.74% ± 16.92% of the total fungal composition (Figure 2F). Venn diagrams depicted the numbers of unique and shared ASVs among different sample groups. The results indicated a higher proportion of unique ASVs in the wild samples (Figure 2A,B).

### 3.3 Comparative Analysis of the Gut Microbiota

The study of the alpha diversity indices of the gut bacterial microbiota in overwintering great bustards revealed that microbial richness (Sobs) ranged from 85 to 403, with the lowest richness observed in sample C1 and the highest in sample W3. The community diversity index (Shannon) ranged from 2.46 to 4.93, with C1 exhibiting the lowest diversity and W3 the highest. The community evenness index ranged from 0.55 to 0.82, with C1 having the lowest evenness and W3 the highest. The coverage index indicated that the community coverage for all samples was greater than 0.99, accurately reflecting the bacterial microbiota composition within the samples (Table S3). Similarly, the study of the alpha diversity indices of the gut fungal microbiota in overwintering great bustards showed that microbial richness (Sobs) ranged from 47 to 191, with the lowest richness in sample W10 and the highest in sample C2. The community diversity index (Shannon) ranged from 1.26 to 4.77, with C3 having the lowest diversity and C2 the highest. The community evenness index ranged from 0.31 to 0.91, with C3 showing the lowest evenness and C2 the highest. The coverage index demonstrated that the community coverage for all samples was greater than 0.99, accurately representing the fungal microbiota composition within the samples (Table S4).

**FIGURE 3:**
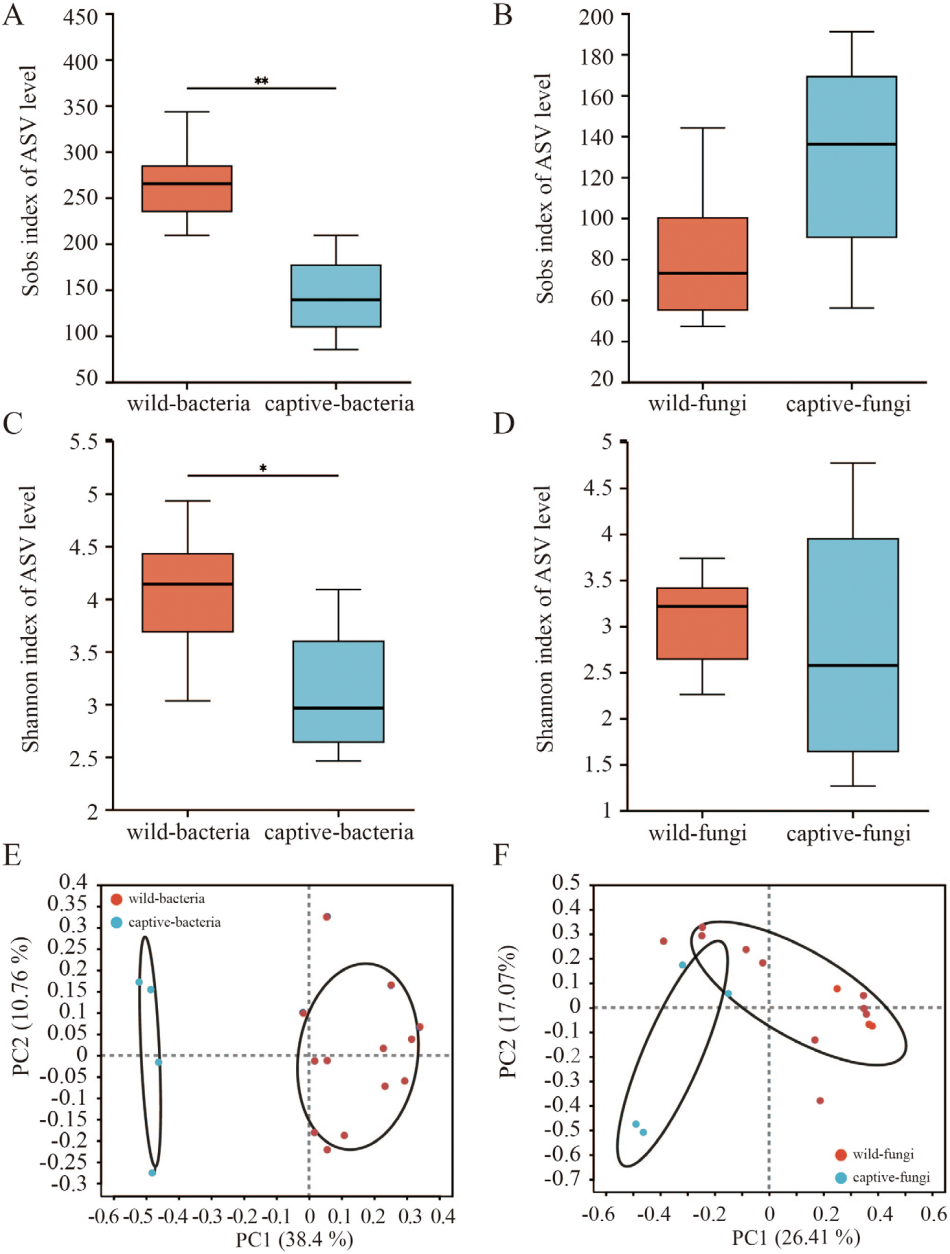
(A) The boxplot illustrates the differences in the Sobs index of the bacterial microbiota between the two sample groups (**p < 0.01). (B) The boxplot illustrates the differences in the Sobs index of the fungal microbiota between the two sample groups. (C) The boxplot illustrates the differences in the Shannon index of the bacterial microbiota between the two sample groups (*p < 0.05). (D) The boxplot illustrates the differences in the Shannon index of the fungal microbiota between the two sample groups. (E) PCoA analysis of bacterial microbiota communities between the two sample groups. (F) PCoA analysis of fungal microbiota communities between the two sample groups.

**FIGURE 4:**
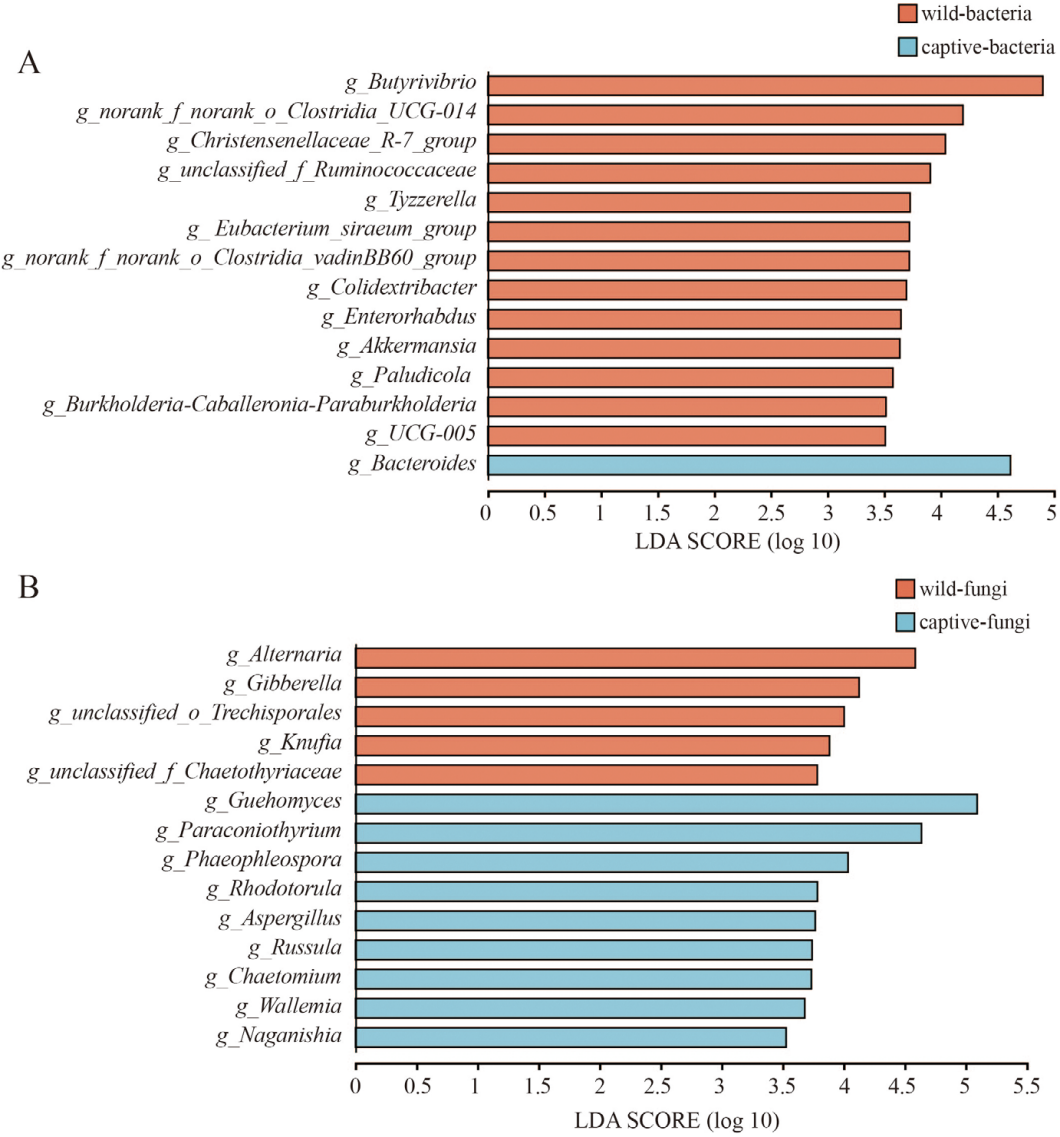
(A) LEfSe analysis was used to identify bacterial taxa in the two sample groups (LDA > 3.5). (B) LEfSe analysis was used to identify fungal taxa in the two sample groups (LDA > 3.5).

**FIGURE 5:**
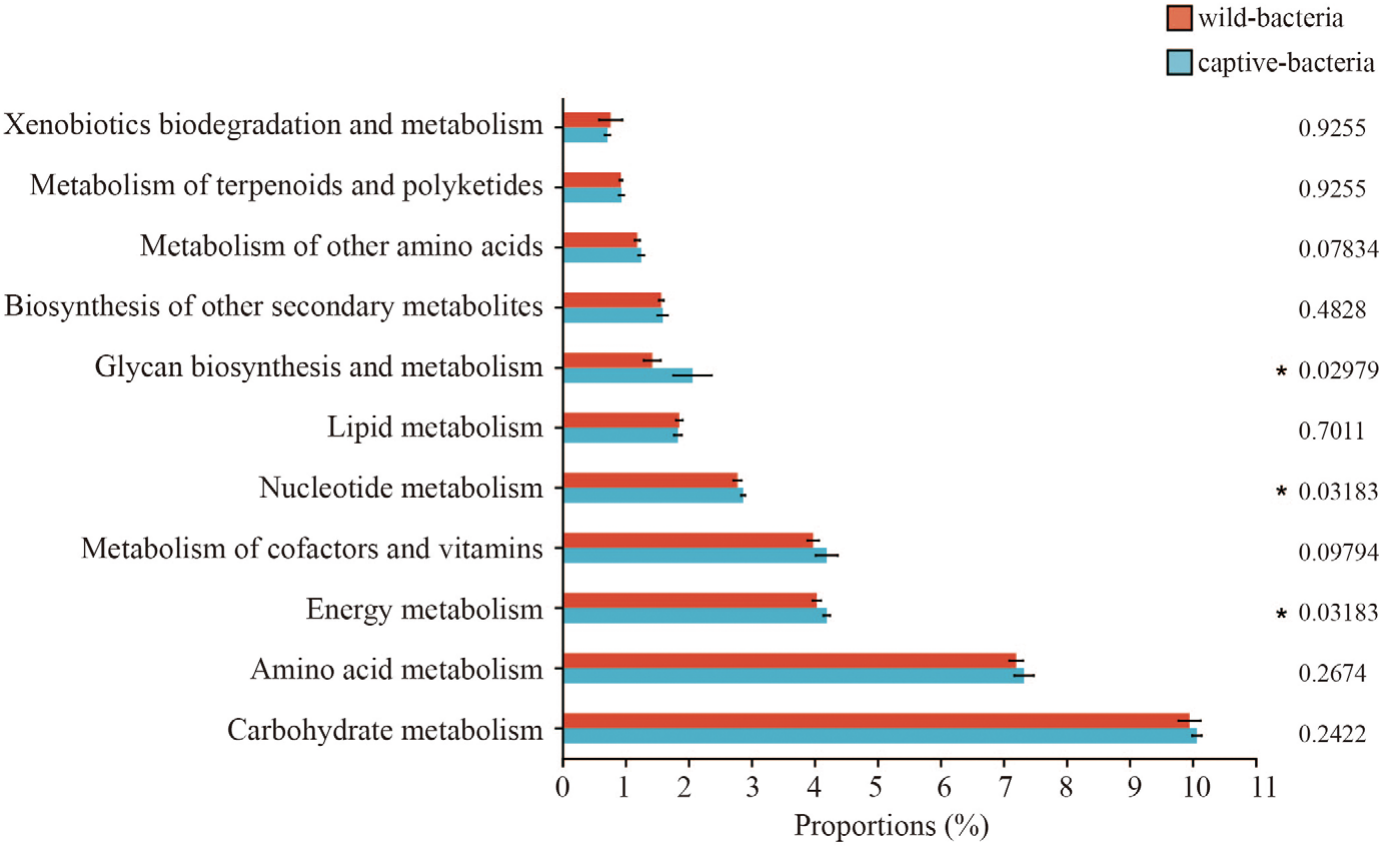
Comparative analysis of the relative abundance of different metabolic pathways in the gut microbiota between the two sample groups (*p < 0.05).

A comparative analysis of alpha diversity indices between the two sample groups revealed that the bacterial microbiota in the wild group exhibited significantly higher richness (Mann–Whitney *U* test: captive vs. wild, *U* = 0.5, *p* = 0.0046, *p*_adjust = 0.0138) and diversity (Mann–Whitney *U* test: captive vs. wild, *U* = 5, *p* = 0.0203, *p*_adjust = 0.0254) compared to the captive group (Figure 3A,C). However, there was no significant difference in the fungal microbiota alpha diversity indices between the two groups (Figure 3B,D). PCoA analysis demonstrated significant differences in the bacterial microbiota composition (PERMANOVA: captive vs. wild, *F* = 8.8159, *R* ^2^ = 0.3702, *p* = 0.003, Figure 3E) and fungal microbiota composition (PERMANOVA: captive vs. wild, *F* = 3.5163, *R* ^2^ = 0.1899, *p* = 0.004, Figure 3F) between the two groups. LEfSe analysis identified 13 bacterial taxa (e.g., *Butyrivibrio*, *norank_f_norank_o_Clostridia_UCG‐014*, *Christensenellaceae_R‐7_group*) in the wild group and 1 bacterial taxon (Bacteroides) in the captive group (Figure 4A). Additionally, 5 fungal taxa (e.g., *Alternaria*, *Gibberella*, *unclassified_o_Trechisporales*) were identified in the wild group, while 9 fungal taxa (e.g., *Guehomyces*, *Paraconiothyrium*, *Phaeophleospora*) were identified in the captive group (Figure 4B). PICRUSt analysis of KEGG level 2 metabolic pathways indicated that the bacterial microbiota in the captive group had significantly higher activity in metabolic pathways related to glycan biosynthesis and metabolism, nucleotide metabolism, and energy metabolism compared to the wild group. No significant differences were observed between the two groups in other metabolic functions (Figure 5).

## 4 Discussion

This study provides the first description of the gut bacterial and fungal microbial composition in caged great bustards, with a comparative analysis conducted against local wild populations. It identifies the differences in gut microbiota composition of great bustards in captive environments.

### 4.1 Changes in Gut Microbiota Composition and Their Ecological Significance

Analysis of the bacterial community composition in great bustard fecal samples reveals that the wild group primarily includes Firmicutes, Bacteroidota, Actinobacteriota, and Proteobacteria, with Firmicutes being the dominant phylum. This composition is consistent with the microbiota found in other wild bird species (Best et al. 2017; Cho and Lee 2020; Fu et al. 2020). In the captive group, the relative abundance of Bacteroidota increases, mirroring the proportion of Firmicutes, while the proportions of Actinobacteriota and Proteobacteria decrease. Similar studies on other bird species, such as *Anser indicus* (Wang et al. 2017) and *Ciconia boyciana* (Wu et al. 2021), also identified Firmicutes as the dominant phylum in both wild and captive populations. However, the relative abundance of Firmicutes is lower in captive populations compared to their wild counterparts.

Research has demonstrated that several genera within the Firmicutes, including *Ruminococcus*, *Lachnospiraceae*, *Butyrivibrio*, and *Christensenellaceae R‐7 group*, possess the ability to degrade plant cellulose, aiding the host in converting indigestible substrates into absorbable short‐chain fatty acids (SCFAs) (Biddle et al. 2013; Flint et al. 2008; Long and Venema 2020; Paillard et al. 2007). SCFAs can be utilized in hepatic gluconeogenesis and lipogenesis (Sun et al. 2017) and are considered important producers of butyrate (Koh et al. 2016; Louis and Flint 2009), which plays a crucial role in maintaining host gut health (Wong et al. 2006). Bacteroidota are also commonly found in herbivorous birds, and several members of the genus *Bacteroidetes* are known to aid in the breakdown of polysaccharides, cellulose, and other complex polymers (Flint et al. 2008; Thomas et al. 2011). Research indicates that consuming legumes can increase the abundance of Bacteroidota while reducing the proportions of Firmicutes and Proteobacteria (Lutsiv et al. 2021). Captive great bustards primarily consume soybeans and peanuts, and this high‐fat, high‐protein diet may induce changes in the gut microbiota composition.

The characterization of the fungal microbiota in great bustard fecal samples indicates that the wild and captive groups have similar microbiota compositions, primarily consisting of Ascomycota and Basidiomycota. This finding is consistent with the fungal community compositions observed in *Anas crecca* (Sakda et al. 2023), *Grus monacha* (Mahtab et al. 2021), and *Anser erythropus* (Liu et al. 2023). The potential functions of Ascomycota and Basidiomycota in avian gut health remain unclear. Previous research has suggested that fungi from the Basidiomycota can produce enzymes necessary for the degradation of plant cellulose, aiding the host in digesting these otherwise indigestible polysaccharides (Arora et al. 2020). Some Ascomycota are known to stimulate the host immune system and combat pathogenic bacteria (Caruffo et al. 2016). During the wintering period, great bustards primarily consume a plant‐based diet, with certain fungal microbes potentially contributing to the degradation of this food. Additionally, the gut fungal microbial community in great bustards exhibits greater stability, which may play a significant role in maintaining gut homeostasis.

### 4.2 Impact of Captive Environment on Gut Microbial Diversity in Great Bustards

Captive animals experience various changes that may impact their gut microbiota, often leading to reduced microbial alpha diversity in some species (Amato et al. 2013; Bahrndorff et al. 2016; Jia et al. 2018; McKenzie et al. 2017). Key factors influencing these changes include alterations in diet and shifts in the environmental microbial landscape (Bornbusch et al. 2024). Results from this study demonstrate a significant decrease in α diversity of bacterial microbiota in the gut of great bustards under captive conditions. Venn diagram results also indicate a reduction in the number of ASVs for both bacteria and fungi in the captive group. Compared to their natural environment, captive great bustards primarily consume homogeneous diets such as soybeans and peanuts, potentially leading to the loss of certain microbial taxa. Furthermore, the habitat environment serves as a reservoir for host‐microbial diversity, and the relatively clean captive environment significantly reduces microbial species diversity, further impacting the diversity of gut microbiota (Reese and Kearney 2019; Sentenac et al. 2022).

LEfSe analysis revealed distinct characteristic taxa in the gut microbiota of great bustards under different environments. In bacterial microbiota, 13 bacterial taxa were identified in wild samples, all belonging to Firmicutes. In contrast, only 1 bacterial taxon belonging to Bacteroidota was identified in captive samples. In fungal microbiota, 5 fungal taxa belonging to Ascomycota were identified in wild samples, whereas 9 fungal taxa belonging to Basidiomycota and Ascomycota were identified in captive samples. These shifts in bacterial and fungal microbiota drove significant changes in β diversity of gut microbiota. However, the consequences of reduced gut microbiota diversity and disrupted β diversity in captive environments for great bustards remain unclear, although several studies have linked dysbiosis to higher disease susceptibility in various animals such as ostriches and chickens (Reese and Kearney 2019; Sentenac et al. 2022).

### 4.3 Prediction of Microbial Functions and Associated Metabolic Pathways

The utilization of the PICRUSt2 algorithm for predicting the metabolic pathways of gut microbiota in great bustards is primarily associated with Carbohydrate metabolism, Amino acid metabolism, Energy metabolism, and Metabolism of cofactors and vitamins. These findings align with the predictive results of metabolic functions of gut microbiota in other wild avian species (Wu et al. 2021; Zhang et al. 2022). Additionally, under captive conditions, there is an enhancement in the host's Energy metabolism, Nucleotide metabolism, and Glycan biosynthesis and metabolism. In natural habitats, overwintering great bustards mainly feed on scattered seeds of maize, wheat, and weeds in farmlands, with a low proportion of high‐protein and high‐fat foods. However, the captive environment's diet rich in fats and proteins may contribute to the observed enhancement in the aforementioned metabolic pathways. Despite PICRUSt2 algorithm's incorporation of larger gene families and reference genome databases, thereby enhancing its accuracy compared to previous methods (Douglas et al. 2020), it still does not encompass the entire metabolic information of the host genome. Future research could utilize metagenomic techniques to delve deeper into the functionality and metabolic pathways of microbial communities and their impact on the health and behavior of great bustards.

## 5 Conclusion

This study provides the first description of the gut bacterial and fungal microbial composition in caged great bustards, with a comparative analysis conducted against local wild populations. Our findings reveal a significant decrease in the α‐diversity of gut microbiota in captive environments, accompanied by notable changes in microbial community structure. Changes in diet and habitat are identified as potential primary drivers of these shifts. Specifically, the artificial feeding of great bustards with high‐fat, high‐protein diets may enhance the nutritional metabolism of their gut microbiota. However, the use of homogeneous feed could lead to the loss of key microbiota, potentially posing health risks.

To improve the likelihood of rescued great bustards successfully returning to the wild, we recommend the following measures: First, establish animal rescue stations close to the wintering grounds of great bustards to preserve the natural environmental conditions as much as possible. Second, thoroughly investigate the natural environment and plant composition at the rescue site to inform the preparation of appropriate food resources for the caged bustards. Third, ensure that rehabilitated great bustards are released back to the rescue site promptly to facilitate their integration with the natural population. While this study offers important insights into the gut microbiota community of captive great bustards, the limited sample size may constrain the generalizability of our results. Future research should encompass a broader range of samples and incorporate more extensive phenotypic analyses to better elucidate the connections between the gut microbiome of great bustards, host health, and environmental adaptation.

## Author Contributions

**Zhiyuan Lu:** data curation (equal), visualization (equal), writing – original draft (equal), writing – review and editing (equal). **Zhucheng Wang:** data curation (equal), visualization (equal), writing – original draft (equal), writing – review and editing (equal). **Hexue Jia:** data curation (equal). **Derong Meng:** data curation (equal), investigation (equal). **Dayong Wu:** conceptualization (equal), writing – review and editing (equal).

## Conflicts of Interest

The authors declare no conflicts of interest.

## Supporting information


Appendix S1.

## Data Availability

The data presented in the study are de posited in the NCBI database (accession numbers: PRJNA 719683, PRJNA 1124359, PRJNA 1124360): accessed via https://www.ncbi.nlm.nih.gov/bioproject/PRJNA719683/ and https://www.ncbi.nlm.nih.gov/bioproject/PRJNA1124359/ and https://www.ncbi.nlm.nih.gov/bioproject/PRJNA1124360/.
