# Supplementary material for: Captivity Reduces Diversity and Shifts Composition of the Great Bustard (Otis tarda dybowskii) Microbiome
Source: Ecol Evol. 2025 Jan 10;15(1):e70836. doi: 10.1002/ece3.70836 (PMC11718221; doi:10.1002/ece3.70836)
Supplement: Supplementary file 1 — Appendix S1. [file ECE3-15-e70836-s001.docx]

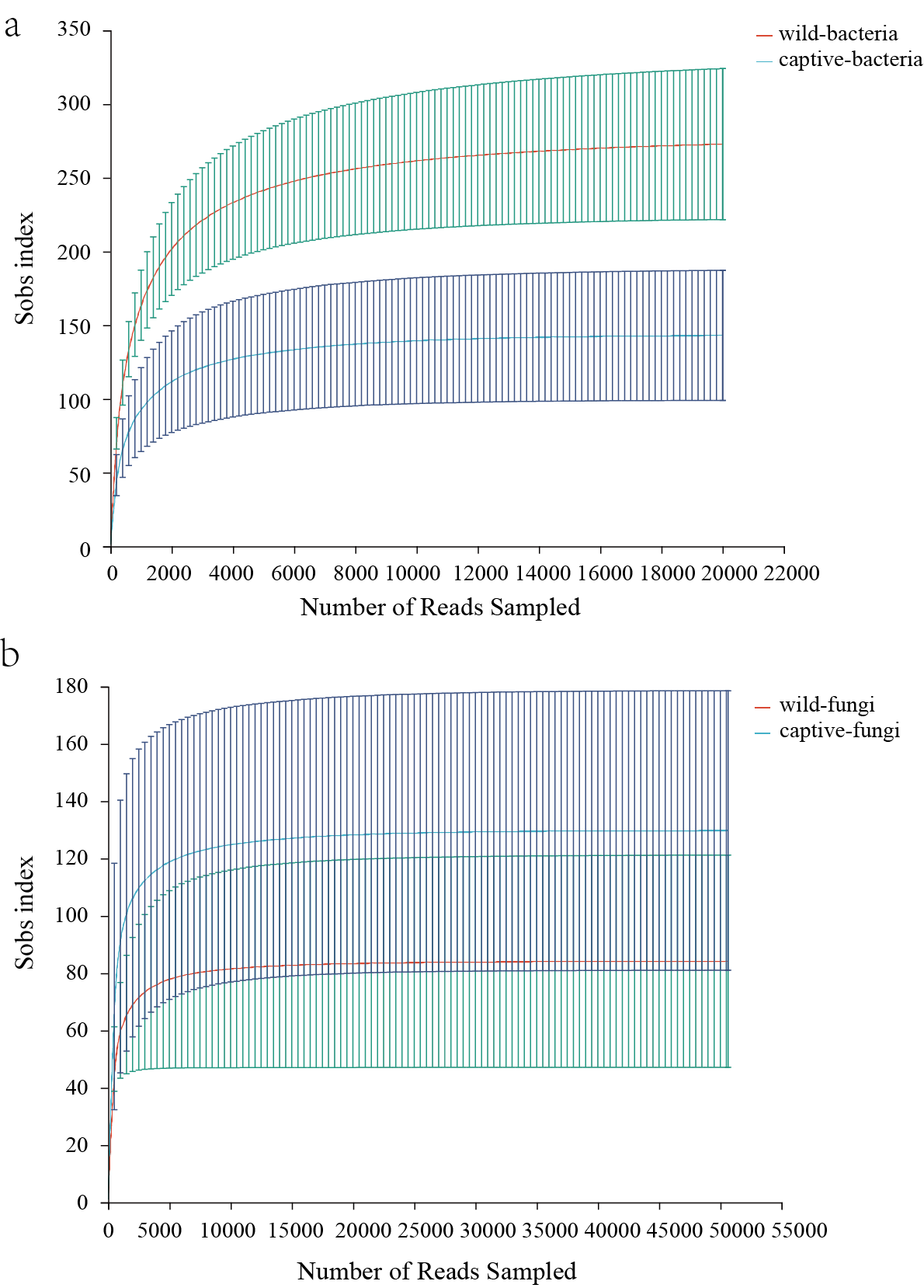


Figure A1. Rarefaction curve depicts the alterations observed in the Sobs index of bacterial microbiota and fungal microbiota in different groups as the sequencing depth is elevated.

Table A1. Statistical table of optimal sequence information of 16s rRNA gene in samples.

| **Sample** | **Seq_num** | **Base_num**  **(bp)** | **Mean_length**  **(bp)** | **Min_length**  **(bp)** | **Max_length**  **(bp)** |
| --- | --- | --- | --- | --- | --- |
| C 1 | 44279 | 18536978 | 418.640394 | 321 | 430 |
| C 2 | 53862 | 22421795 | 416.282258 | 265 | 442 |
| C 3 | 49062 | 20028056 | 408.219314 | 391 | 511 |
| C 4 | 42665 | 17840027 | 418.141966 | 337 | 431 |
| W 1 | 53112 | 21707385 | 408.709614 | 239 | 430 |
| W 2 | 49887 | 20405319 | 409.03079 | 254 | 497 |
| W 3 | 55913 | 22773493 | 407.302291 | 221 | 500 |
| W 4 | 57545 | 23528123 | 408.864767 | 276 | 431 |
| W 5 | 44502 | 18039801 | 405.370568 | 270 | 511 |
| W 6 | 62311 | 25433715 | 408.173757 | 231 | 479 |
| W 7 | 60368 | 24542914 | 406.555029 | 245 | 490 |
| W 8 | 55849 | 22687419 | 406.227847 | 305 | 489 |
| W 9 | 45015 | 18377260 | 408.247473 | 232 | 490 |
| W 10 | 41065 | 16801609 | 409.146694 | 262 | 430 |
| W 11 | 49779 | 20254151 | 406.881436 | 336 | 488 |
| W 12 | 43874 | 17980010 | 409.810138 | 214 | 430 |
| W 13 | 40785 | 16761428 | 410.970406 | 248 | 434 |

Table A2. Statistical table of optimal sequence information of ITS gene in samples.

| **Sample** | **Seq_num** | **Base_num**  **(bp)** | **Mean_length**  **(bp)** | **Min_length**  **(bp)** | **Max_length**  **(bp)** |
| --- | --- | --- | --- | --- | --- |
| C 1 | 81260 | 19686908 | 242.270588 | 147 | 522 |
| C 2 | 65644 | 15632242 | 238.136646 | 141 | 471 |
| C 3 | 57305 | 13462192 | 234.921769 | 156 | 531 |
| C 4 | 70013 | 16302174 | 232.844957 | 143 | 524 |
| W 1 | 73968 | 17052727 | 230.541951 | 143 | 499 |
| W 2 | 58311 | 14110849 | 241.992917 | 150 | 427 |
| W 3 | 58329 | 14009935 | 240.188157 | 181 | 461 |
| W 4 | 69793 | 16348158 | 234.237789 | 162 | 488 |
| W 5 | 65700 | 16275328 | 247.721887 | 178 | 479 |
| W 6 | 70397 | 15670889 | 222.607341 | 145 | 414 |
| W 7 | 74545 | 16363154 | 219.507063 | 141 | 527 |
| W 8 | 62847 | 15133608 | 240.800802 | 191 | 522 |
| W 9 | 69700 | 15305212 | 219.586973 | 165 | 409 |
| W 10 | 57939 | 15131937 | 261.170144 | 141 | 426 |
| W 11 | 74326 | 15685199 | 211.032465 | 177 | 522 |
| W 12 | 70757 | 15603060 | 220.516133 | 142 | 527 |
| W 13 | 70932 | 15556451 | 219.314992 | 181 | 410 |

Table A3. Bacterial microbial alpha diversity index for each sample.

| **Sample** | **Sobs** | **Shannon** | **Shannoneven** | **Coverage** |
| --- | --- | --- | --- | --- |
| C 1 | 85 | 2.458422 | 0.553368 | 1 |
| C 2 | 209 | 4.087146 | 0.765049 | 0.99975 |
| C 3 | 134 | 2.817725 | 0.575299 | 1 |
| C 4 | 144 | 3.10488 | 0.624748 | 1 |
| W 1 | 259 | 4.261225 | 0.766845 | 0.9994 |
| W 2 | 265 | 3.487264 | 0.624988 | 0.99935 |
| W 3 | 403 | 4.928899 | 0.821629 | 0.998851 |
| W 4 | 235 | 4.427121 | 0.81089 | 0.9998 |
| W 5 | 259 | 3.312805 | 0.596168 | 0.9995 |
| W 6 | 277 | 4.579254 | 0.814232 | 0.9994 |
| W 7 | 224 | 3.030398 | 0.559977 | 0.99995 |
| W 8 | 295 | 3.685884 | 0.648127 | 0.998851 |
| W 9 | 278 | 4.139376 | 0.735546 | 0.99965 |
| W 10 | 343 | 4.110457 | 0.704119 | 0.9991 |
| W 11 | 216 | 4.109285 | 0.764478 | 0.9998 |
| W 12 | 209 | 4.322538 | 0.80911 | 0.9999 |
| W 13 | 284 | 4.538834 | 0.803479 | 0.99975 |

Table A4. Fungal microbial alpha diversity index for each sample.

| **Sample** | **Sobs** | **Shannon** | **Shannoneven** | **Coverage** |
| --- | --- | --- | --- | --- |
| C 1 | 147 | 3.129612 | 0.627122 | 0.99998 |
| C 2 | 191 | 4.767554 | 0.907712 | 1 |
| C 3 | 56 | 1.263522 | 0.313891 | 1 |
| C 4 | 125 | 2.014376 | 0.417201 | 1 |
| W 1 | 172 | 3.685666 | 0.716012 | 0.999961 |
| W 2 | 55 | 3.660068 | 0.913343 | 1 |
| W 3 | 67 | 3.735904 | 0.888508 | 1 |
| W 4 | 73 | 3.213761 | 0.749048 | 0.99998 |
| W 5 | 48 | 3.318223 | 0.857156 | 1 |
| W 6 | 105 | 2.257078 | 0.48498 | 1 |
| W 7 | 100 | 2.515277 | 0.546185 | 0.99998 |
| W 8 | 57 | 3.397538 | 0.84034 | 1 |
| W 9 | 144 | 3.105115 | 0.624795 | 1 |
| W 10 | 47 | 3.41065 | 0.885849 | 1 |
| W 11 | 97 | 2.757058 | 0.602674 | 1 |
| W 12 | 75 | 2.511863 | 0.581788 | 1 |
| W 13 | 54 | 2.641893 | 0.662297 | 1 |
